# The Aldehyde Dehydrogenase ALDH2*2 Allele, Associated with Alcohol Drinking Behavior, Dates Back to Prehistoric Times

**DOI:** 10.3390/biom11091376

**Published:** 2021-09-17

**Authors:** Chih-Lang Lin, Rong-Nan Chien, Li-Wei Chen, Ting-Shuo Huang, Yu-Chiau Shyu, Chau-Ting Yeh, Kung-Hao Liang

**Affiliations:** 1 Liver Research Unit, Keelung Chang Gung Memorial Hospital, Keelung 204, Taiwan; wn49792000@yahoo.com.tw (C.-L.L.); leiwei@cgmh.org.tw (L.-W.C.); 2 Liver Research Center, Chang Gung Memorial Hospital, Taoyuan 333, Taiwan; ronald@adm.cgmh.org.tw; 3 Community Medicine Research Center, Keelung Chang Gung Memorial Hospital, Keelung 204, Taiwan; huangts@adm.cgmh.org.tw (T.-S.H.); yuchiaushyu@gmail.com (Y.-C.S.); 4 College of Medicine, Chang Gung University, Taoyuan 333, Taiwan; 5 Department of General Surgery, Chang Gung Memorial Hospital, Keelung 204, Taiwan; 6 Department of Nursing, Chang Gung University of Science and Technology, Taoyuan 333, Taiwan; 7 Institute of Molecular Biology, Academia Sinica, Taipei 115, Taiwan; 8 Department of Medical Research, Taipei Veterans General Hospital, Taipei 112, Taiwan; 9 Institute of Food Safety and Health Risk Assessment, National Yang-Ming Chiao-Tung University, Taipei 112, Taiwan; 10 Institute of Biomedical Informatics, National Yang-Ming Chiao-Tung University, Taipei 112, Taiwan

**Keywords:** aldehyde dehydrogenase, haplotype, metabolic disorder, gene-behavior interactions, germline variant

## Abstract

Human alcohol-consumption behavior is partly genetically encoded. The alcohol consumption of 987 residents in Keelung, Taiwan, was evaluated by using the Alcohol Use Disorder Identification Test (AUDIT). We assessed ~750,000 genomic variants of 71 residents who drank hazardously (AUDIT score ≥ 8) and 126 residents who did not drink in their daily lives (AUDIT score = 0), using high-density single nucleotide polymorphism (SNP) arrays. The rs671 G > A manifests the highest significance of the association with drinking behavior (Fisher’s exact P = 8.75 × 10^−9^). It is a pleiotropic, non-synonymous variant in the aldehyde dehydrogenase 2 (ALDH2) gene. The minor allele “A”, commonly known as ALDH2*2, is associated with non-drinkers. Intriguingly, identity-by-descent haplotypes encompassing genomic regions with a median length of 1.6 (0.6–2.0) million nucleotide bases were found in all study participants with either heterozygous or homozygous ALDH2*2 (*n* = 81 and 13, respectively). We also analyzed a public-domain dataset with genome-wide genotypes of 2000 participants in Guangzhou, a coastal city in Southern China. Among them, 175 participants have homozygous ALDH2*2 genotype, and again, long ALDH2*2-carrying haplotypes were found in all 175 participants without exceptions. The median length of the ALDH2*2-carrying haplotype is 1.7 (0.5–2.8) million nucleotide bases. The haplotype lengths in the Keelung and Guangzhou cohorts combined indicate that the origin of the ALDH2*2 allele dates back to 7935 (7014–9381) years ago. In conclusion, the rs671 G > A is the leading genomic variant associated with the long-term drinking behavior among residents of Keelung, Taiwan. The ALDH2*2 allele has been in Asian populations since prehistoric times.

## 1. Introduction

Inventions of alcoholic beverages predate written history. Evidence of the earliest manmade alcoholic beverages, which date back to 9000 years ago, was found in the Henan province of China [1]. Since then, alcohol-containing beverages have made profound social, economic and health impacts across the globe [2,3,4,5,6,7,8]. Alcohol can produce personally varied euphoria [9]. Human cognitive and sensory capabilities are also reduced under the influence of alcohol. The euphoria and other physiological effects altogether determine habitual drinking behaviors. Alcohol-use disorder is developed in some persons, causing burdens to families and societies. This disorder is influenced by genetics, with 60% heritability estimated in a twin study [10]. To pinpoint genetic variants associated with habitual drinking behaviors, many candidate-gene and genome-wide association studies (GWAS) have been conducted [11,12,13,14,15,16,17]. Interestingly, implications of drinking-behavior-related neurological and psychiatric genes remain unclear despite these explorations. Rather, genomic variants of metabolic genes, such as alcohol dehydrogenases (ADHs) and aldehyde dehydrogenases (ALDHs), have been reported frequently [11,14,15], suggesting the intimate connections between alcohol metabolisms and drinking behaviors. These genomic variants dictate personal metabolic rates of ethanol and aldehydes, including acetaldehyde [18] and 4-HNE [19]. A recent pan-ethnic group study in conjunction with a meta-analysis showed that the single nucleotide polymorphism (SNP) rs671, located at exon 12 of the ALDH2 gene, has the strongest association in East Asians [11].

Moreover, rs671 G > A is a non-synonymous, Asian-specific [20] genomic variant. The minor allele “A”, also known as the ALDH2*2 allele, encodes a glutamate-to-lysine substitution at the position 487 of the mature protein (Glu487Lys), which effectively disrupts enzymatic activities (Online Mendelian Inheritance in Man (OMIM) #100650) and causes the accumulation of aldehydes in the human body [21]. Apart from alcohol consumptions, this pleiotropic variant is associated with traits such as alcohol-induced facial flushing after drinking [22], blood pressure [23], chronic heart disease [24,25], post-stroke epilepsy [19], late-onset Alzheimer’s disease [26], fasting plasma glucose levels [27], gout [28], hip fracture and osteoporosis [29], lumbar disc herniation [30], breast cancer in postmenopausal Asian women [31], esophageal cancer [32] and hepatocellular carcinoma [33]. Considering the detrimental effects of ALDH2*2 on the enzymatic functions, which are apparently essential to humans, it remains mysterious why this allele persists in Asian populations without being purged by purifying selections. To date, the time of emergence of the ALDH2*2 allele continues to be elusive, apart from one reported value of 2000~3000 years ago shown in the literature [21,34,35,36,37]. This value first appeared in a study which investigated only five local genomic variants surrounding rs671 [34].

Community-based studies are investigations of the general populations in certain defined geographical regions. Community-based studies are suitable for investigating issues pertaining to food, lifestyle, culture, behavior patterns and occupations. The Keelung City, located in the northern coast of Taiwan, is a historic harbor city once colonized by the Spanish, Dutch and Japanese. Keelung is at a pivotal location connecting East and Southeast Asia and has been the gateway of international trades for centuries. The distance from Keelung to Mainland China is ~180 km across the Taiwan Strait. The population size is ~370,000, of whom most are of Chinese ancestry. We investigated the alcohol consumption demographics of Keelung residents by using the original, unmodified definition of the Alcohol Use Disorder Identification Test (AUDIT) score developed by the World Health Organization (WHO) in 1993 [38]. Based on prior statistics in Taiwan and worldwide, AUDIT scores ≥ 8 were indicative of hazardous drinking [39]. Thus, residents with the AUDIT scores ≥ 8 were selected for a genome-wide comparison with controls who did not drink in their daily life.

## 2. Materials and Methods

### 2.1. Study Subjects

This study was approved by the institutional review board of Chang Gung Memorial Hospital, Taiwan, and conducted according to the ethical guidelines of the 1975 Declaration of Helsinki. Written informed consents were obtained from all participants in this community study. All of the study subjects were adults at the time of enrollment. An AUDIT questionnaire in Chinese was used, which was directly translated from the WHO AUDIT questionnaire without modifications [38]. The standard drink is defined as 10 g, consistent with WHO [38] but lower than the definition in USAUDIT (i.e., 14 g), which is optimized for residents in United States [40]. In this community study, 987 participants were enrolled into the Northeastern Taiwan Community Medicine Research Cohort (NTCMRC, ClinicalTrials.gov Identifier: NCT04839796) between February and November 2019, and screened using the AUDIT questionnaire. Among them, 24 individuals were found to drink extremely heavily, reaching the criteria of AUDIT score ≥ 16 (Supplementary Materials Figure S1). Additionally, 194 individuals were found to have 8 ≤ AUDIT score < 16. We selected all 24 patients with the AUDIT score ≥ 16, and randomly selected 47 patients with 8 ≤ AUDIT score < 16 as the heavy-drinker group in this case-control study. Most of these 71 patients are male (91.55%). To select suitable non-drinker controls for a genome-wide comparison, we performed the propensity score matching by using two basic demographic variables, namely age and gender, in the case:control ratio of 1:2, in participants who did not drink in their daily life (AUDIT score = 0). This procedure was performed by using the “MatchIt” package of the R statistical software. A total of 142 control samples were found matched with case patients. We then randomly selected 126 patients from the 142 patients as the non-drinker group, due to the remaining processing capacity of our microarray plates, each of which can handle 96 samples. The case (71 individuals who drank heavily; AUDIT score ≥ 8) and the control (126 residents who did not drink at all; AUDIT score = 0) participants were selected for a subsequent genome-wide comparison. Demographic parameters were documented and evaluated, including gender, age, BMI, coffee- and tea-consumption behavior, education levels and exercise habits (Table 1). Peripheral bloods were collected from these participants and centrifuged for separating the blood cells from plasma. Total DNA was extracted from the peripheral blood cells for subsequent genome-wide genotyping.

### 2.2. Genome-Wide Genotype Calling and Assessment of Associations

The Affymetrix Axiom Genome-Wide TWB 2.0 array plates, designed jointly by the Taiwan Biobank, the National Center for Genome Medicine in Taiwan and Thermo Fisher Scientific (Waltham, MA, USA), were used for assessing the genome-wide variants. Genome-wide genotyping was performed by the National Center for Genome Medicine in Taiwan, where a rigorous quality-control pipeline has been installed to process the data before release. The Affymetrix APT base-calling software was used. Associations between genotypes and phenotypes were evaluated by using the Fisher’s exact test, based on major and minor allele counts in case and control subjects. We used the Golden Helix SVS software v.8.8.3 for genome-wide comparisons of genotypes (Golden Helix, London, UK).

### 2.3. Obtaining Genotypes from a Public-Domain Database to Augment This Study

We searched the European Nucleotide Archive (https://www.ebi.ac.uk/eva/?Home accessed on 9 September 2021), an important public-domain database of genomic variants. We found the genome-wide genotypes of a participant cohort (ID: PRJEB42554, published 21 January 2021) which are useful for the augmentation of this study. This cohort has a decent sample size (*n* = 2000) with participants recruited in Guangzhou, a coastal city in Southern China. The genotypes were assayed by using SNP arrays. We downloaded the genotypes in the format of compressed VCF files, where the SNP locations have been annotated based on the GRCh37 reference genome. The numbers of participants with rs671 “GG”, “GA” and “AA” genotypes are 937, 888 and 175, respectively. The high number of “AA” carriers was an important merit which motivated us to choose this dataset. The minor allele frequency is 30.95%.

### 2.4. Obtaining Allele-Carrying Haplotypes from Densely Genotyped Persons

Allele-carrying haplotypes in individuals with homozygous alleles at a variant site were first searched. Starting from the closest variant at both sides of the allele, the haplotype was extended at both the upstream and downstream directions, one at a time, as long as the genotype of the next variant was still homozygous and was identical to the genotype of all the other individuals (Supplementary Materials Figure S2). A core region of haplotype was found when the consecutive genotypes identical across all the individuals in both upstream and downstream directions were linked together. The disagreement of genotypes in different persons began to emerge at the boundary of the core region. The haplotypes were extended further as long as this individual who has the homozygous allele in the next variant, which was also the major allele in the remaining members. Otherwise, individuals with the other allele were designated to be no longer extending in this direction, and their haplotype boundary was found. When the number of people with the “extending” status was reduced to 2, and the subsequent variants of the two people differed, so that no major allele could be determined, the extending process in this direction stopped. Finally, the longest haplotype joining the left and right arms was found. This longest haplotype provided a template for phasing the individuals with the heterozygous allele. The algorithm for finding allele-carrying haplotypes in persons with the homozygous allele are presented in a flowchart in Supplementary Materials Figure S2.

### 2.5. Mutation Age Estimation Using Haplotype Length Distributions

The age of the ALDH2*2 allele founder was estimated by using the gamma method, utilizing the linkage-disequilibrium decay effect caused by prior recombination events [41,42,43]. This method is based on the premise that the allele-carrying haplotypes are copies of the ancient chromosome where the founder allele emerges. The haplotype initially encompasses the entire haploid chromosome, yet is gradually shortened by the subsequent recombination events, causing a linkage-disequilibrium decay [41,42]. Nevertheless, the remaining allele-carrying haplotypes still harbor hundreds of variants genotyped in a dense genotyping microarray, indicating identity-by-descent. The lengths offer a means of calculating the ages. The shorter the haplotype is, the older the founder allele and allele-carrying haplotype are.

To estimate the time, the genomic locations of the haplotype boundaries were first cross-referenced with the genetic map offered by the international HapMap project for quantifying the haplotype lengths in the units of Morgans. The lengths would manifest a gamma distribution defined by a shape parameter and a scale parameter (α,τ). The shape parameter is set as 2 [42]. The number of generations (τ) is the scale parameter, which can be calculated by using the maximum-likelihood estimations with a bias-correction [42] defined in Equation (1):

(1) $$\tau =\frac{2n-1}{\sum_{i=1}^{n}l_{i}}$$

where the $l_{1},\;l_{2},\;l_{3}\ldots ,\;l_{n}$ are the haplotype lengths in the unit of Morgans; *n* is the number of haplotypes investigated. The age of the founder mutation is then inferred from the number of generations, using the time per generation, which is assumed to be 25 years in this study:

Age = (time per generation) × (τ)(2)

We wrote a Python script implementing the above calculations in Equations (1) and (2) with a bootstrapping method [44]. The script was executed in the Python 3.6 environment. Bootstrapping is a random sampling with replacement method that, for each step of sampling, *n* haplotypes were drawn from the pool of *n* haplotypes, regardless of whether the haplotype have or have not been drawn, for calculating one age estimate. The sampling process was repeated to produce more age estimates, creating a distribution of estimated ages.

## 3. Results

### 3.1. The Genomic Variant rs671 G > A Is the Leading Variant Associated with Alcohol Consumption Behavior

**Figure 1:**
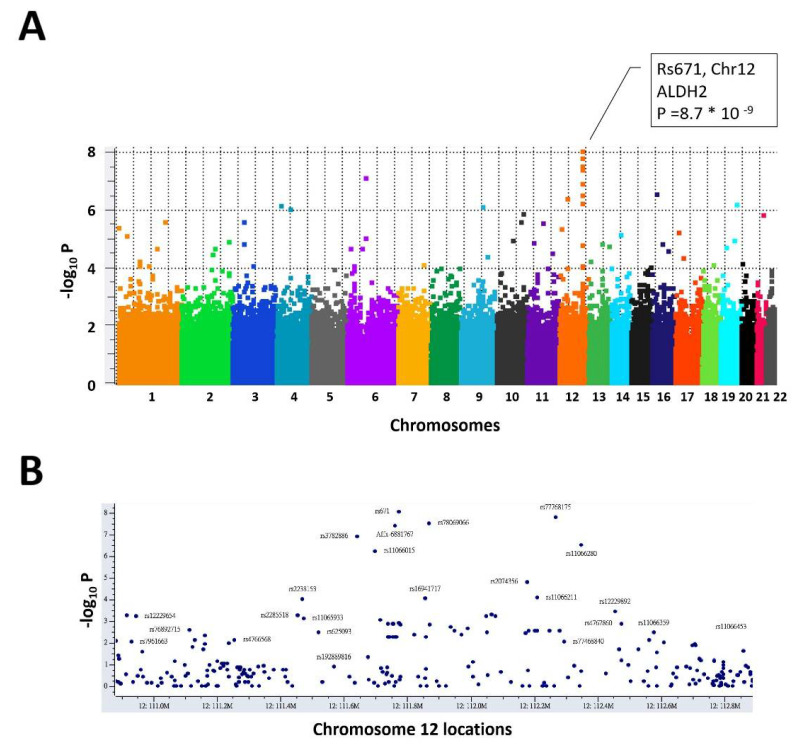
Genome-wide comparisons of residents in Keelung who drank hazardously and who did not drink in their daily life. (**A**) Manhattan plot of autosomal chromosomes. (**B**) Zoom-in plot of association in the vicinity of rs671 (2 million nucleotide bases; Chr12:110,912,851-112,927,816 in GRCh38).

We assessed ~750,000 SNPs in 197 residents in Keelung, including 71 heavy drinkers (AUDIT score ≥ 8) and 126 non-drinkers (AUDIT score = 0). The two groups did not manifest disparity in terms of gender, age, body mass index (BMI), coffee- and tea-consumption behavior, education levels and exercise frequencies [41] (Table 1). Among all genomic variants, rs671 G > A in chromosome 12 manifested the strongest association (P = 8.75 × 10^−9^, Figure 1A). There are 103, 81 and 13 persons with the GG, GA and AA genotypes at rs671, respectively (Table 2). The minor allele A, also known as the ALDH2*2, is associated with non-drinkers, and the minor allele frequency (MAF) is 0.272. None of the heavy drinkers had the homozygous “AA” genotype. All the variants with *p*-values smaller than 5 × 10^−7^ are summarized in Table 2. The genotype counts of all these variants do not deviate from Hardy–Weinberg equilibrium. In Table 2, the variants in the vicinity of rs671 showed similar genotype counts, reflecting the linkage disequilibrium effects among them. A zoom-in Manhattan plot of this neighborhood showed a typical multiple-variant association in the presence of linkage disequilibrium (Figure 1B).

### 3.2. A Haplotype Shared by All ALDH2*2-Carrying Individuals Indicates Its Identity by Descent

**Figure 2:**
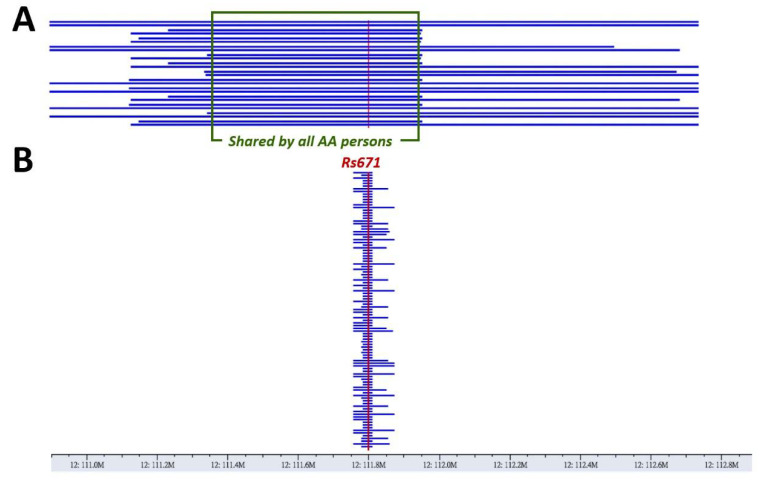
The haplotypes inferred in the vicinity of rs671 (Chr12:110,912,851-112,927,816), shown as blue horizontal lines. The vertical red lines depict the spot of rs671. (**A**) The rs671 “A”-allele-carrying haplotypes in persons with homozygous “AA” genotypes. Each person has two haplotypes shown as two consecutive blue horizontal lines (13 persons, 26 haplotypes). In each aligned genomic location of the blue horizontal lines, the nucleotide bases are identical, without exceptions. Within the green box, all persons have identical haplotypes encompassing 38 consecutive variants. (**B**) The rs671 “G”-allele-carrying haplotypes, calculated by using the same method as in (**A**), in persons with homozygous “GG” genotypes.

Identical haplotypes encompassing 138 consecutive, densely genotyped genomic variants, spanning a region of ~600,000 nucleotide bases surrounding rs671, were found, with no exceptions, in all 13 persons with homozygous ALDH2*2 (shown within the green rectangular box of Figure 2A). In this region, genotypes of all variants are consistently homozygous, showing clearly the existence of two identical haplotypes, each of which carries one ALDH2*2 allele. This region is referred to as the “core” region of the haplotype. The “core” haplotype can be extended further in some but not all 13 persons, and it can finally encompass a maximum of 439 consecutive genomic variants, spanning a genomic region of two million nucleotide bases (Chr12:110,912,851-112,927,816 in GRCh38, Figure 2A). The longest haplotype represents a replica of an ancient chromosomal segment where the “G” allele at this mutation site changed to “A”. According to the linkage-disequilibrium decay theory, the longer the haplotype is, the younger the allele is [41,42,43]. We performed the same analysis in 103 residents with the homozygous “GG” genotypes for a comparison. Much shorter shared haplotypes were observed (Figure 2B). This suggests an earlier emergence of the “G” allele. These allele-carrying haplotypes in residents with rs671 “AA” genotype could be identical by descent, considering the hundreds of identical variant types shared by all people. 

The longest haplotype observed in residents with rs671 “AA” also provided a reference template for haplotype phasing of the 81 residents with the “GA” genotype. The median length of all the ALDH2*2-carrying haplotypes found in the Keelung cohort is 1.6 million nucleotide bases, corresponding to 0.361 centiMorgans in this chromosomal region. The minimum and maximum lengths are 0.6 and 2.0 million bases. 

We also analyzed the genome-wide genotypes of the Guangzhou cohort, particularly the 175 participants with homozygous ALDH2*2 genotypes. Long ALDH2*2-carrying haplotypes were found in all 175 participants without exceptions. The median (min, max) lengths is 1.7 (0.5–2.8) million nucleotide bases. The fact that all of the homozygous AHDH2*2 participants in Keelung and Guangzhou have ALDH2*2-carrying haplotypes longer than 0.5 million bases supports the idea that this allele has one single origin. This allele is less likely to have arisen recurrently at different ancient chromosomes.

### 3.3. Age of the ALDH2*2 Allele Was Estimated to Be 7935 Years Ago

**Figure 3:**
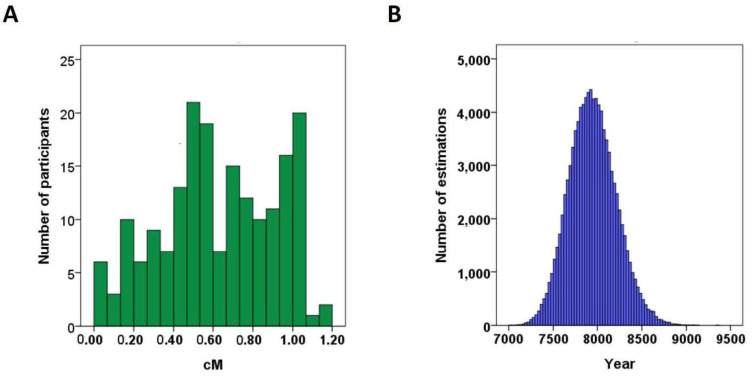
(**A**) Histogram of lengths of ALDH2*2 haplotypes in centiMorgans of the participants with homozygous ALDH2*2 in the Keelung and Guangzhou cohorts (*n* = 183). (**B**) Histogram of the estimated age (years) of ALDH2*2 allele by 100,000 bootstrapping estimations. The minimum, median and maximum of this distribution are 7014, 7935 and 9381 years, respectively. The arithmetic mean of the distribution is 7944 years ago.

The left and right boundaries of the haplotypes represent the historical recombination sites. Based on the lengths of the ALDH2*2-carrying haplotypes between the two boundaries, the age of the most recent common ancestor can be inferred [42]. To ensure an adequate sample size for age estimation, participants with the homozygous ALDH2*2 allele in the Keelung and Guangzhou cohorts (*n* = 13 and 175 respectively) were included and combined together. We then transformed the physical lengths of these haplotypes into genetic lengths in the units of centiMorgans (cM), utilizing the map of conversion offered by the international HapMap project. The length distribution of these ALDH2*2-carrying haplotypes is shown in Figure 3A. Subsequently, we made 100,000 bootstrap estimations of the age, and they are shown in Figure 3B. In this distribution, the median age is 7935 (min–max; 7014–9381) years ago.

## 4. Discussion

Young-age alleles tend to have small minor allele frequencies (e.g., <0.01) in a population, as time is required for them to populate [41]. Compared with common alleles, variants of such small MAFs are less likely to be detected in a typical GWAS, where variants are assessed in the same way with a fixed sample size regardless of their wide diversity in MAFs [45]. Overall, rs671 is a common variant in Asia. A recent study revealed the wide geographical distribution of this allele in East and Southeast Asia [46]. The distribution centered in the Fujian and Guangdong, two coastal provinces of China, where the MAF could be as high as 0.409 and 0.357, respectively [46]. Similar distributions were shown in one other study, where the region with the highest MAF was assumed the geographical origin of ALDH2*2 [34]. The authors further inferred the time origin of this allele by using the time of emergence of the “Pai-Yuei” ethnic people who have populated this geographical region since 2000–3000 years ago [34]. This value was subsequently cited in the literature [21,35,36,37]. However, the region with the highest MAF does not always co-localize with the geographical origin. Thus, the assumption behind the estimation of 2000–3000 years may be undermined. In human migratory history, a small subset from a large genetically diverse population may migrate to and then populate a new territory. The minor alleles enriched in the immigrants may cause a sudden increase of corresponding MAFs. This is called the founder effect. The wide geographical distribution and the high MAF of ALDH2*2 prompted us to ask whether this allele could have such a short history.

Thus, we investigated the allele-carrying haplotypes by using our densely genotyped data, which are critical for inferring the historical genomic recombination events surrounding the allele. As people migrated, they took the alleles and the haplotypes along. We discovered an identity-by-descent allele-carrying haplotype spanning 138 consecutive, densely genotyped genomic variants, covering ~600,000 nucleotide bases in the vicinity of rs671 in the Keelung cohort. The median haplotype length is 1.6 (0.6–2.0) million nucleotide bases. We also utilized a public-domain dataset of Guangzhou, the capital of the Guangdong province where the ALDH2*2 frequency is high. Again, we found long ALDH2*2-carrying haplotypes in all 175 participants with the homozygous ALDH2*2 allele without exceptions. The median haplotype length is 1.7 (0.5–2.8) million nucleotide bases. In contrast, the previous study [34] only employed five local variants spanning a genomic region of 40,000 nucleotide bases, which would not be sufficient to offer high-resolution time estimates. We had two discoveries. First, an identity-by-descent relatively long ALDH2*2-carrying haplotype was found consistently in all investigated individuals with this allele. Second, the ALDH2*2 was ascertained to have arisen in a pre-historical, possibly Neolithic time of 7935 years ago, which matched the time of the earliest known alcohol-containing beverages in China [1]. What we have estimated is the most recent common ancestor of the included individuals with ALDH2*2. The birth of the allele might have happened at an even more ancient time. The ALDH2*2 allele was not observed in the residents of the Americas; thus, it is reasonable to infer that the allele did not emerge before humans emigrated from Asia to the Americas through the Bering sea ~16,500 years ago [47,48].

After the emergence of this allele, it stably persisted, rather than have come and gone, in Asian populations. It is still mysterious why this enzymatic-function disrupting allele was not purged by purifying selection. One possibility is that the enzyme dysfunction produced a face flushing and discomfort phenotype of the allele carriers whenever they drank, notifying them to stop drinking excessively. The consequences of this behavior counteracted purifying selections.

## 5. Conclusions

The SNP rs671 G > A is the leading genomic variant associated with the drinking behavior of people in Keelung, Taiwan. The origin of the ALDH2*2 allele dates back to 7935 years ago.

## Figures and Tables

**Table 1:** Demographic information of subjects in this genome-wide association study.

	AUDIT Score ≥ 8	AUDIT Score = 0	P
Patient number	71	126	
Gender			
Male	65 (91.55%)	114 (90.48%)	0.802 *
Female	6 (8.45%)	12 (9.52%)	
Age, years, Mean ± SD	53.72 ± 9.93	56.97 ± 12.53	0.062 ^‡^
BMI, kg/m^2^, Mean ± SD	25.6 ± 4.0	25.8 ± 3.7	0.643 ^‡^
<24,	25 (35.2%)	41 (32.5%)	0.597 *
24–27	24 (33.8%)	37 (29.4%)	
>27	22 (31.0%)	48 (38.1%)	
Coffee			
never	24 (33.8%)	36 (28.6%)	0.483 *
social	19 (26.8%)	44 (34.9%)	
more than sometimes	28 (39.4%)	46 (36.5%)	
Tea			
never	15 (21.1%)	36 (28.6%)	0.401 *
social	24 (33.8%)	44 (34.9%)	
more than sometimes	32 (45.1%)	46 (36.5%)	
Education level			
No education	1 (1.4%)	4 (3.2%)	0.609 ^‡^
Elementary school	8 (11.3%)	23 (18.3%)	
Junior high school	15 (21.1%)	19 (15.1%)	
Senior high school	34 (47.9%)	34 (27.0%)	
College	11 (15.5%)	38 (30.2%)	
Higher education	2 (2.8%)	8 (6.3%)	
Exercise			
little	23 (33.8%)	41 (31.9%)	0.699 *
Sometimes (≤2 days per week)	17 (25.0%)	26 (23.7%)	
Frequent (≥3 days per week)	28 (41.2%)	59 (44.4%)	

* X^2^ tests; ^‡^ Welsh *t*-tests.

**Table 2:** Leading variants associated with alcohol consumption levels in this GWAS.

SNP	Chr	Gene	Allele	Case			Control			MAF	Fisher’s Exact P	Hardy–Weinberg Equilibrium P	
				Homozygous Major	Heterozygous	Homozygous Minor	Homozygous Major	Heterozygous	Homozygous Minor			Case	Control
rs116369005	6	Intergenic	G/C	38	26	0	113	7	0	0.090	7.54 × 10^−8^	0.125	0.947
rs671	12	ALDH2	G/A	56	15	0	47	66	13	0.272	8.75 × 10^−9^	0.609	0.345
rs77768175	12	HECTD4	A/G	56	15	0	48	65	13	0.269	1.56 × 10^−8^	0.609	0.416
rs78069066	12	MAPKAPK5/TMEM116	G/A	54	17	0	45	68	13	0.282	2.96 × 10^−8^	0.519	0.225
rs3782886	12	BRAP	T/C	53	18	0	46	67	13	0.282	1.20 × 10^−7^	0.473	0.282
rs11066280	12	HECTD4	T/A	52	18	1	43	70	13	0.294	3.05 × 10^−7^	0.923	0.136
rs541300736	16	TEKT5	G/A	36	25	0	112	8	0	0.091	2.62 × 10^−7^	0.132	0.931

## Data Availability

The genome-wide genotypes of 197 study subjects will be provided as a CSV file by the co-corresponding authors to academic scientists on reasonable request.

## Supplementary Materials

The following are available online at https://www.mdpi.com/article/10.3390/biom11091376/s1. Supplementary Figure S1. The AUDIT score distribution of screened participants in this community study. Supplementary Figure S2. The algorithm used for exploring the allele carrying haplotypes in a group of people with the homozygous allele.
