# Supplementary material for: The Aldehyde Dehydrogenase ALDH2*2 Allele, Associated with Alcohol Drinking Behavior, Dates Back to Prehistoric Times
Source: Biomolecules. 2021 Sep 17;11(9):1376. doi: 10.3390/biom11091376 (PMC8465343; doi:10.3390/biom11091376)

Article

The Aldehyde Dehydrogenase ALDH2*2 Allele, Associated with Alcohol Drinking Behavior, Dates Back to Prehistoric Times

Chih-Lang Lin [1,2,3,4], Rong-Nan Chien [2,3,4], Li-Wei Chen [1,3,4], Ting-Shuo Huang [3,4,5], Yu-Chiau Shyu [3,6,7], Chau-Ting Yeh [2,4,*] and Kung-Hao Liang [8,9,10,*]

Supplementary Figure S1. The AUDIT score distribution of screened participants in this community study.

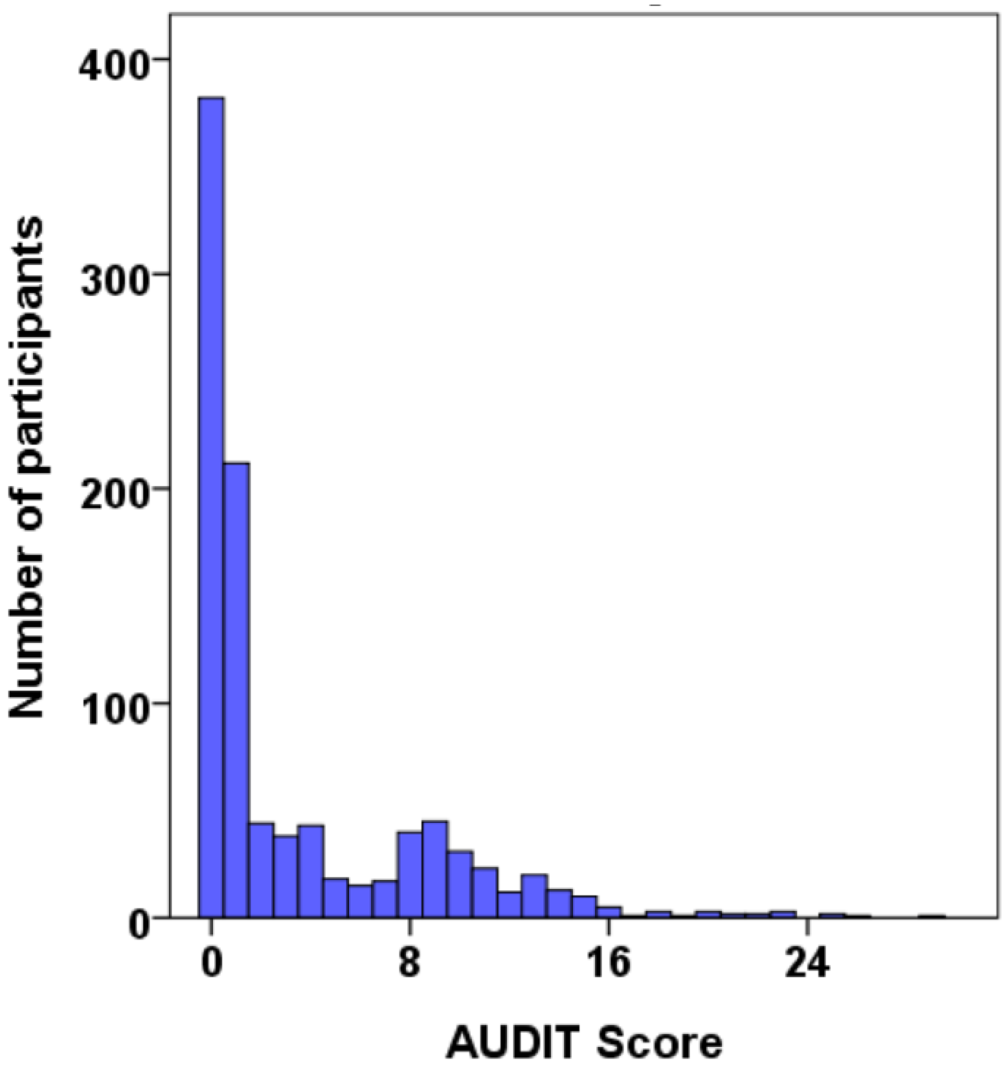

Supplementary Figure S2. The algorithm used for exploring the allele carrying haplotypes in a group of people with the homozygous allele.

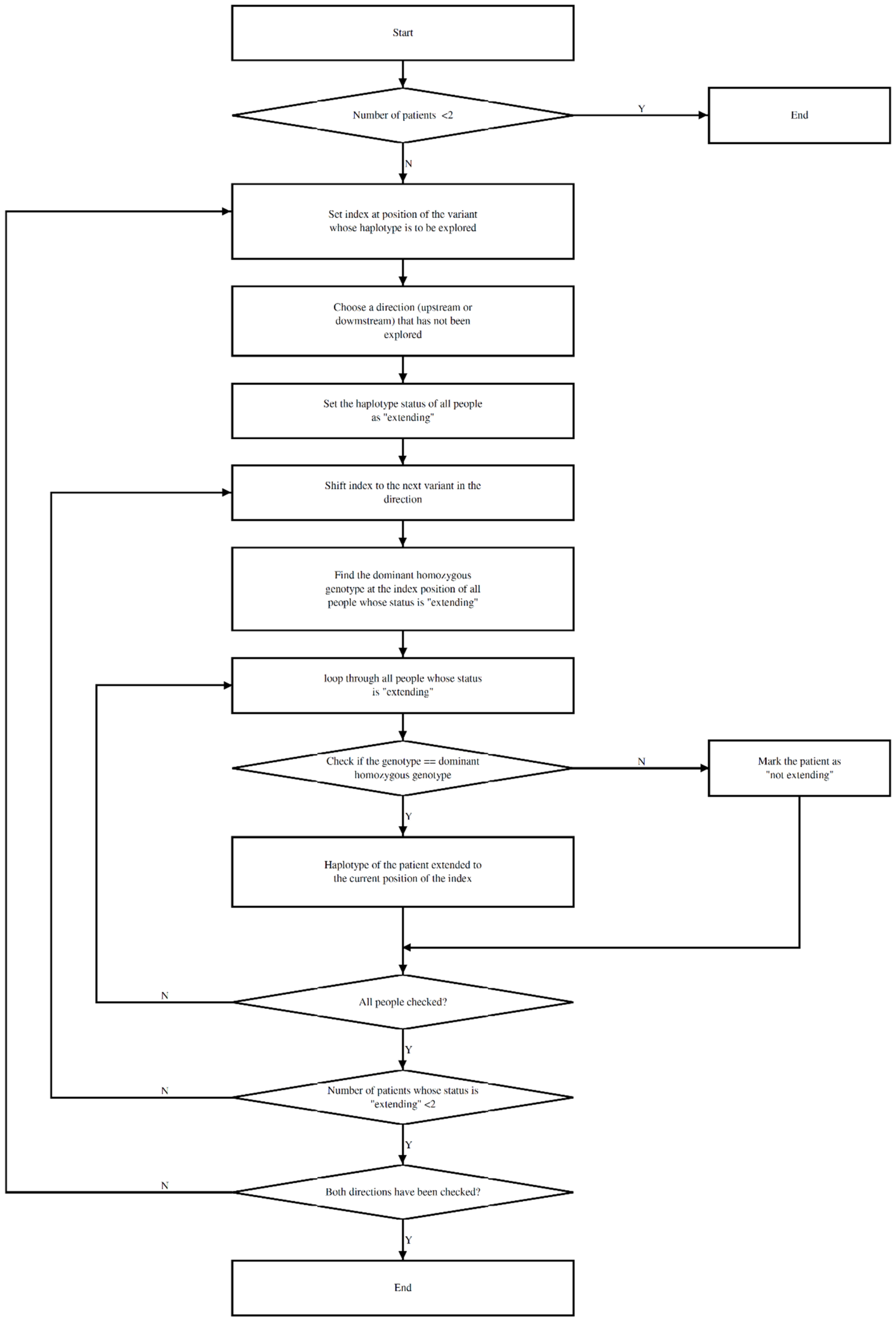

Supplement: Supplementary file 1 [file biomolecules-11-01376-s001.zip › biomolecules-1364791-supplementary.pdf]
